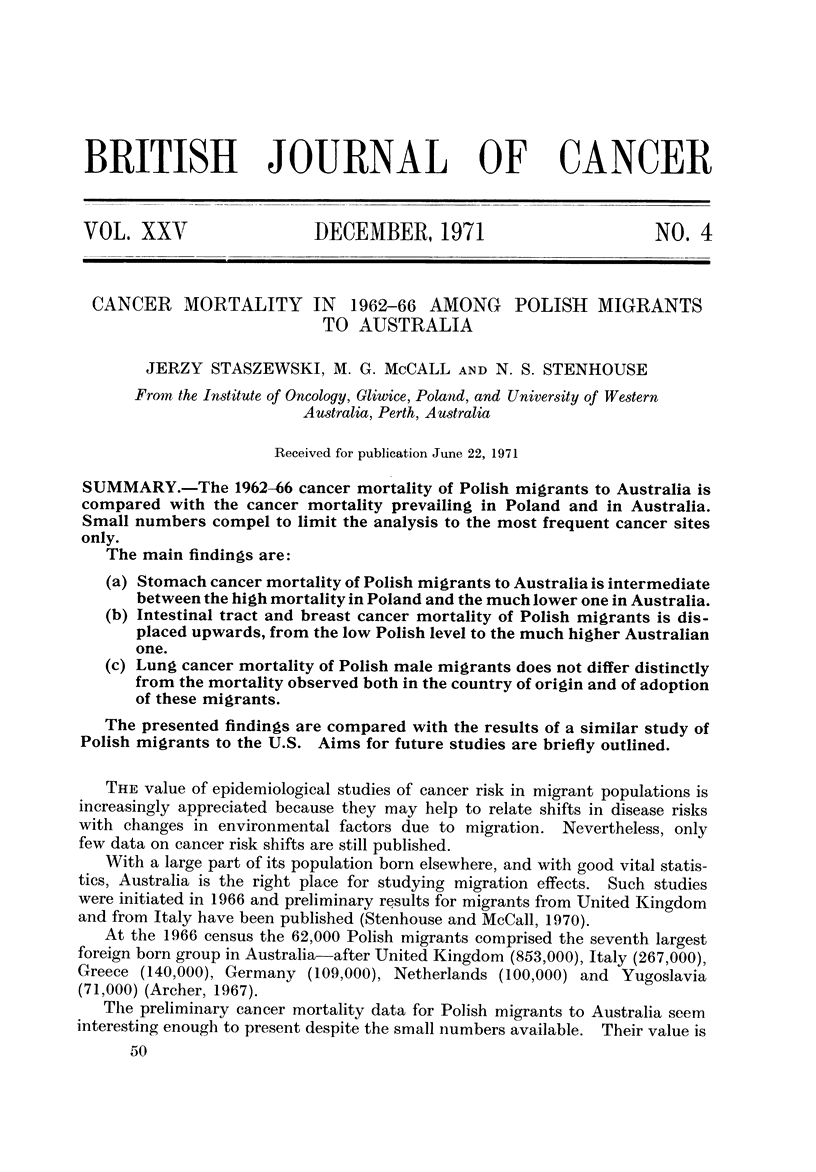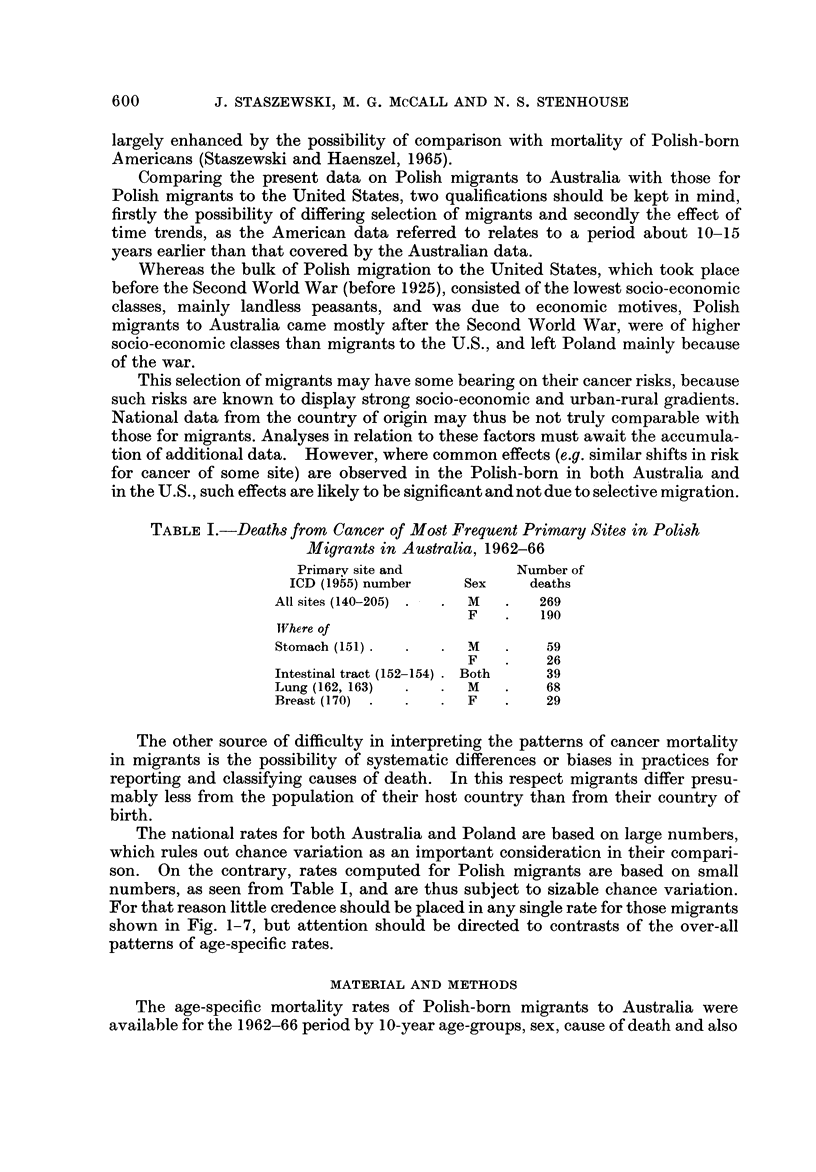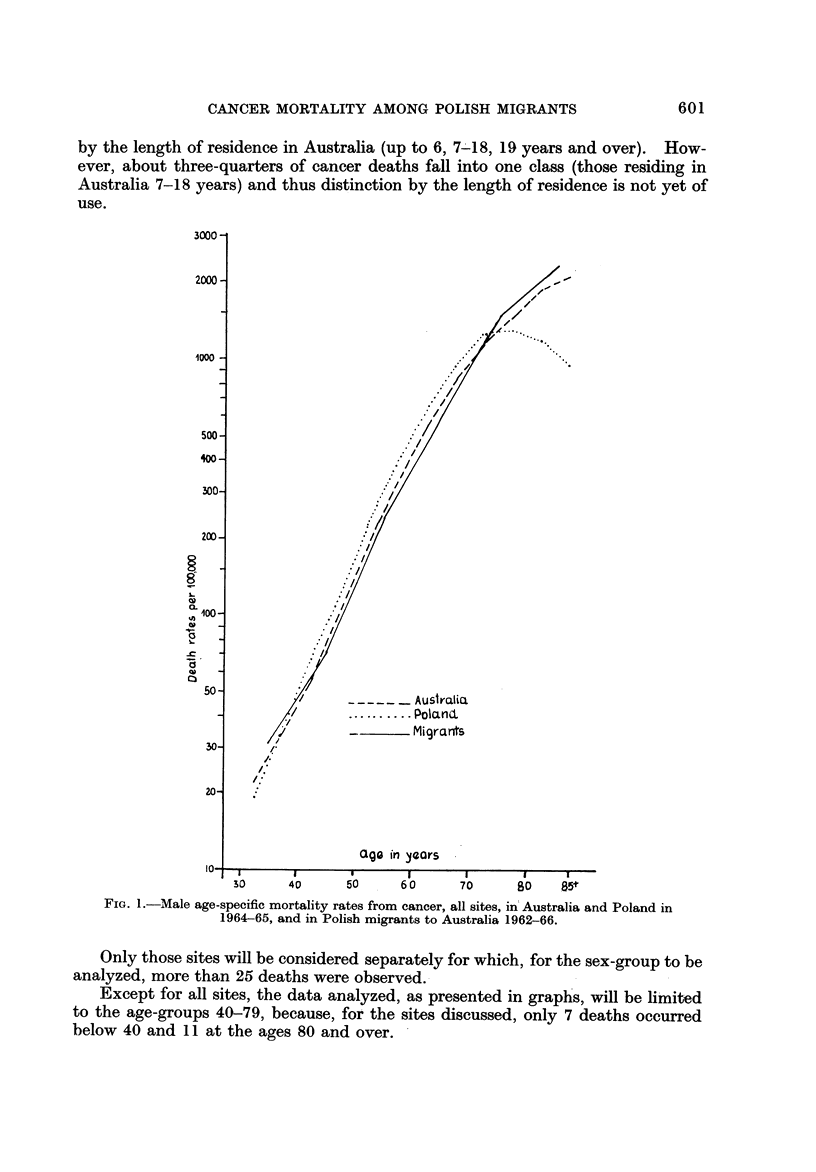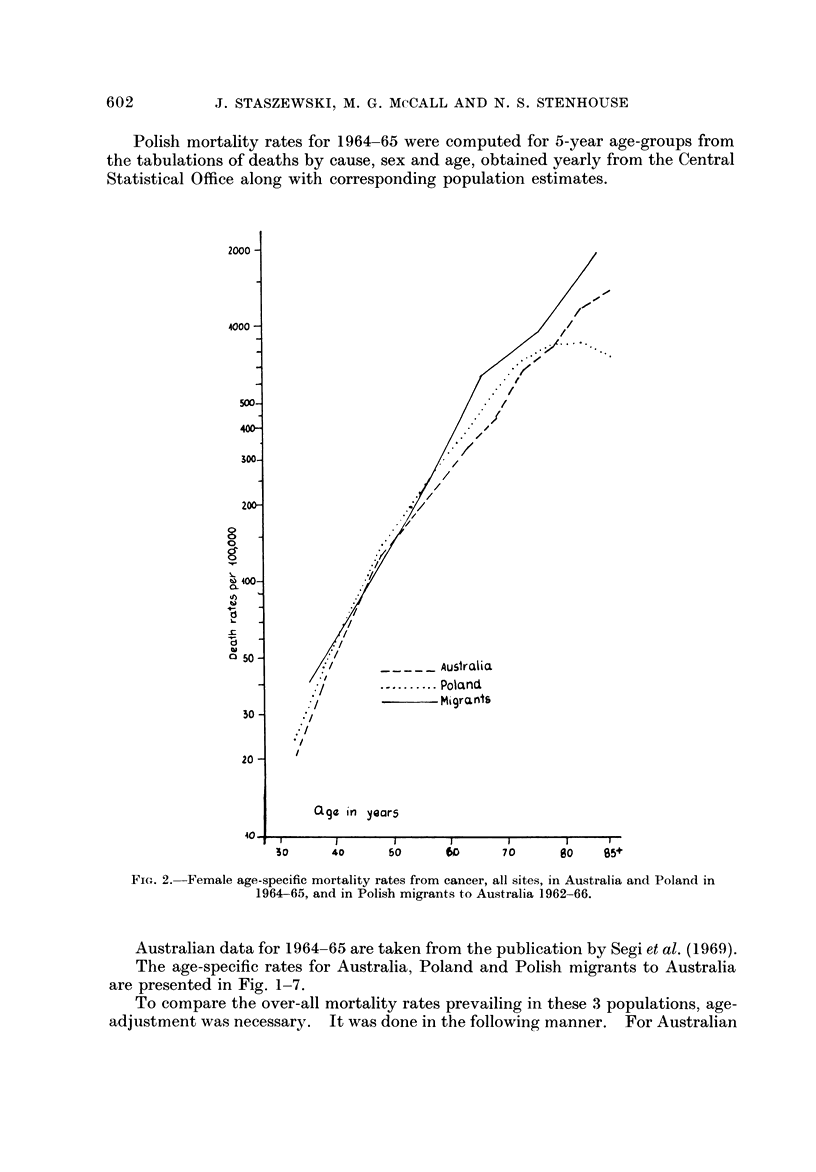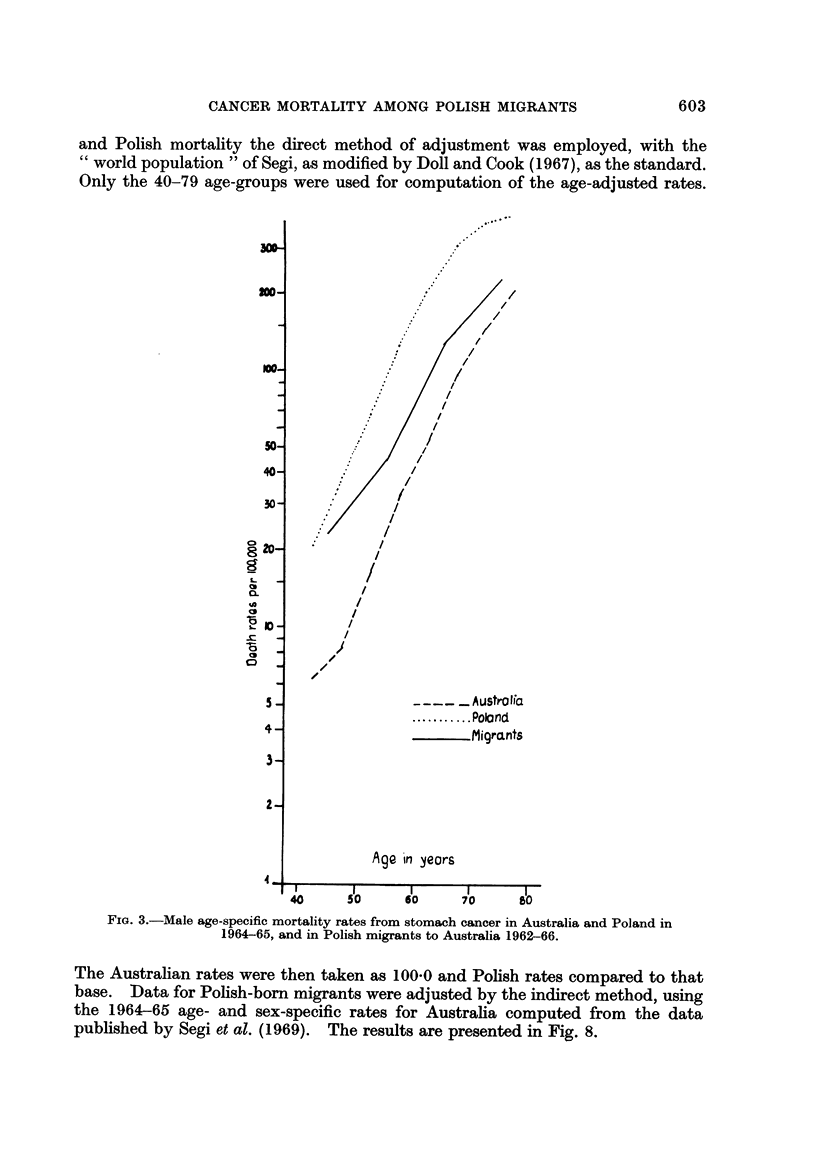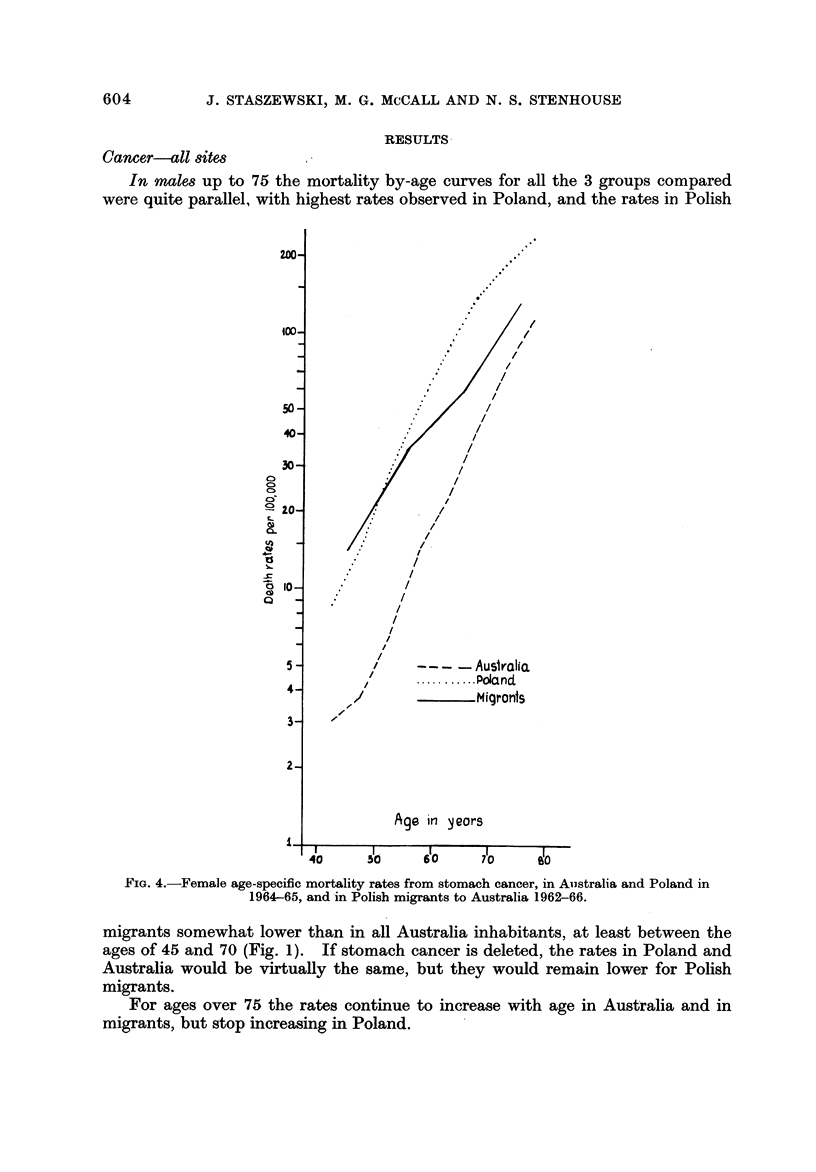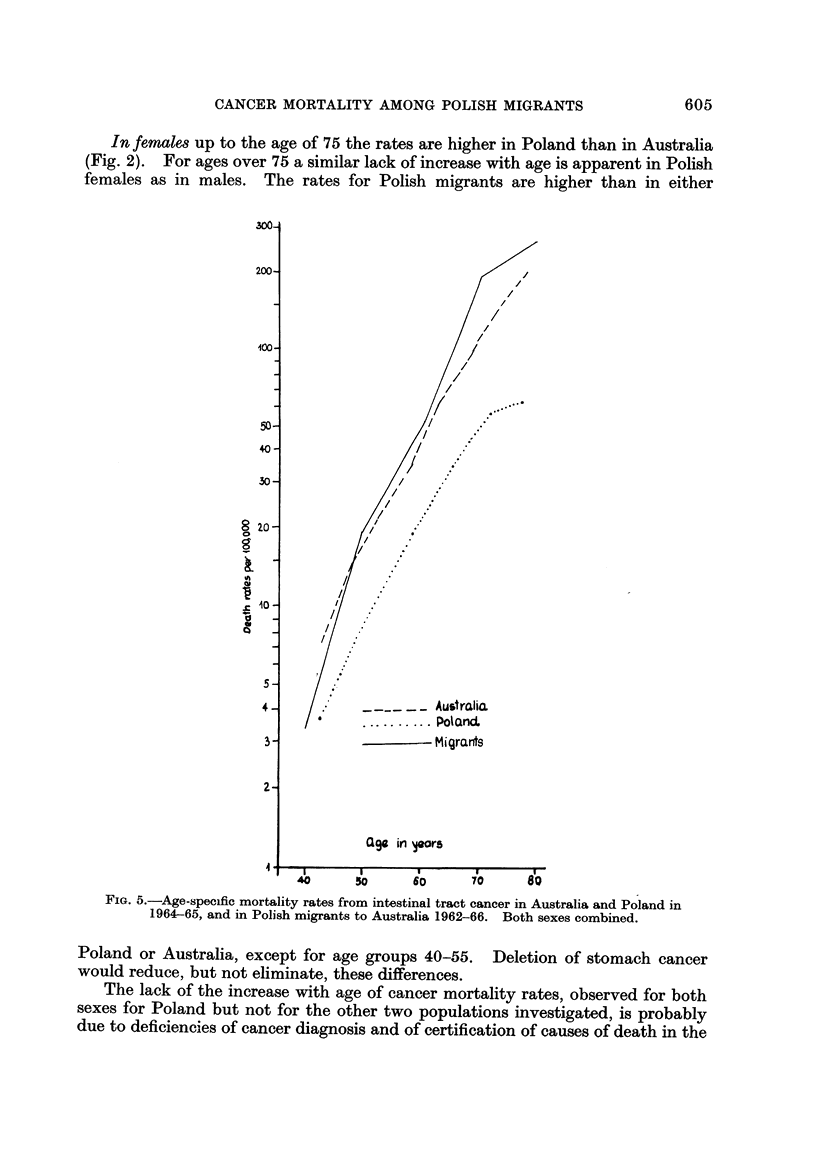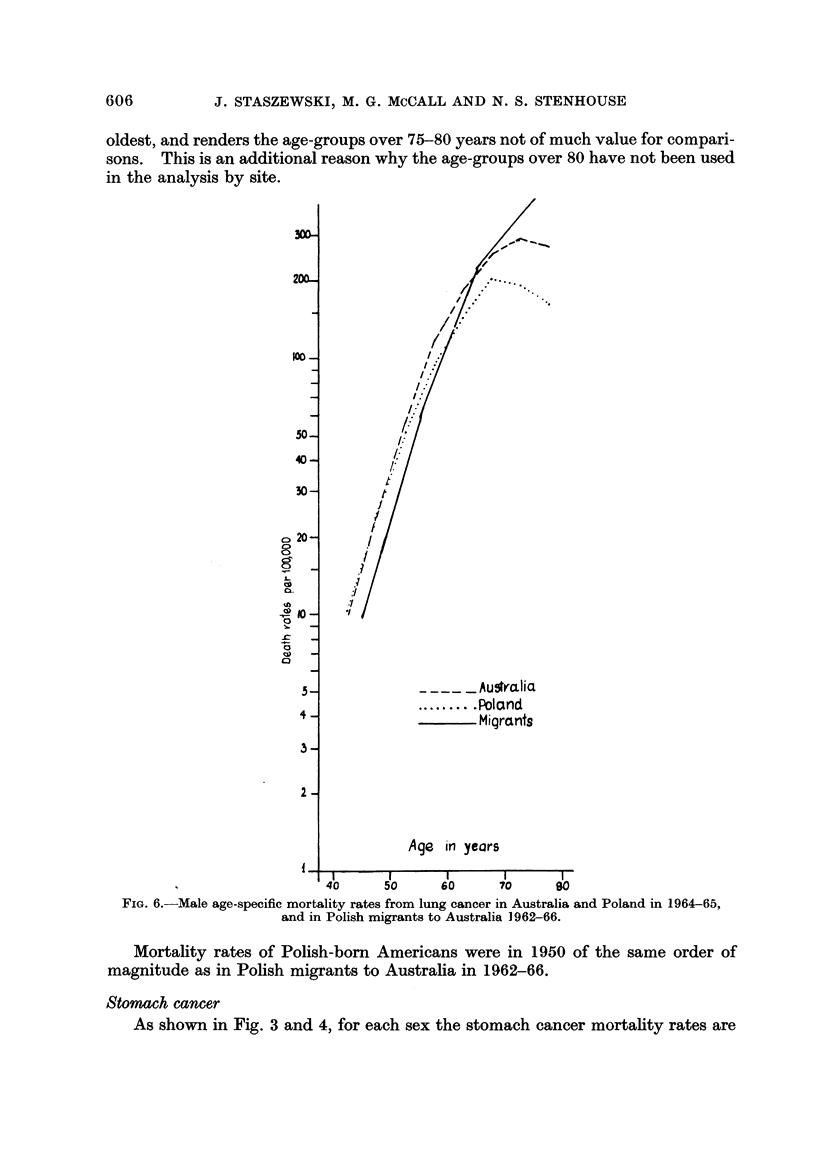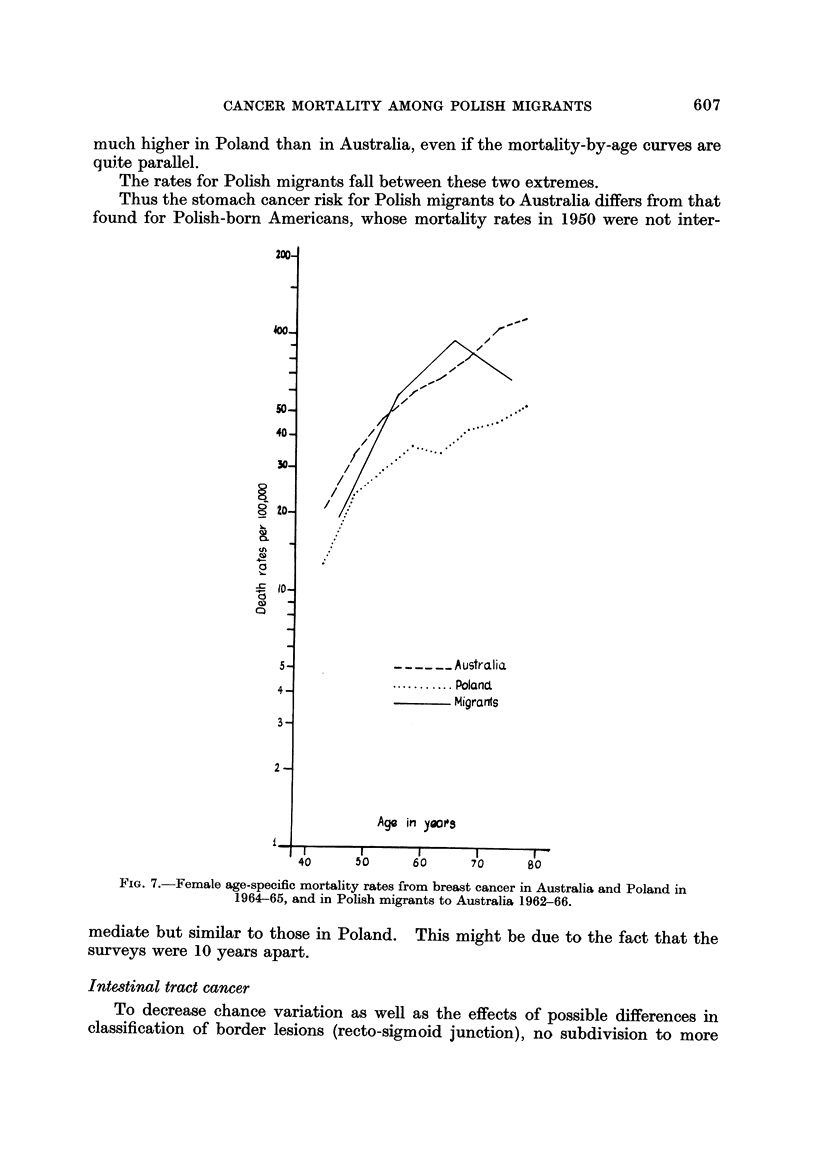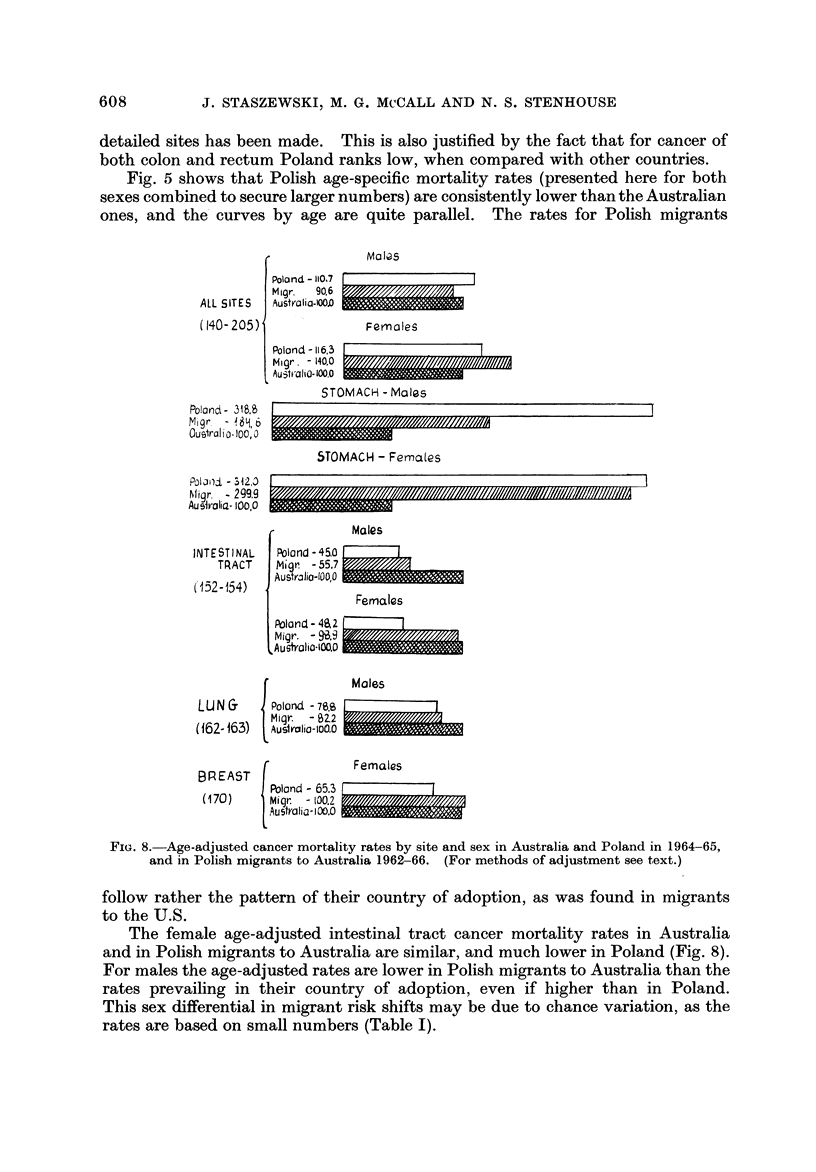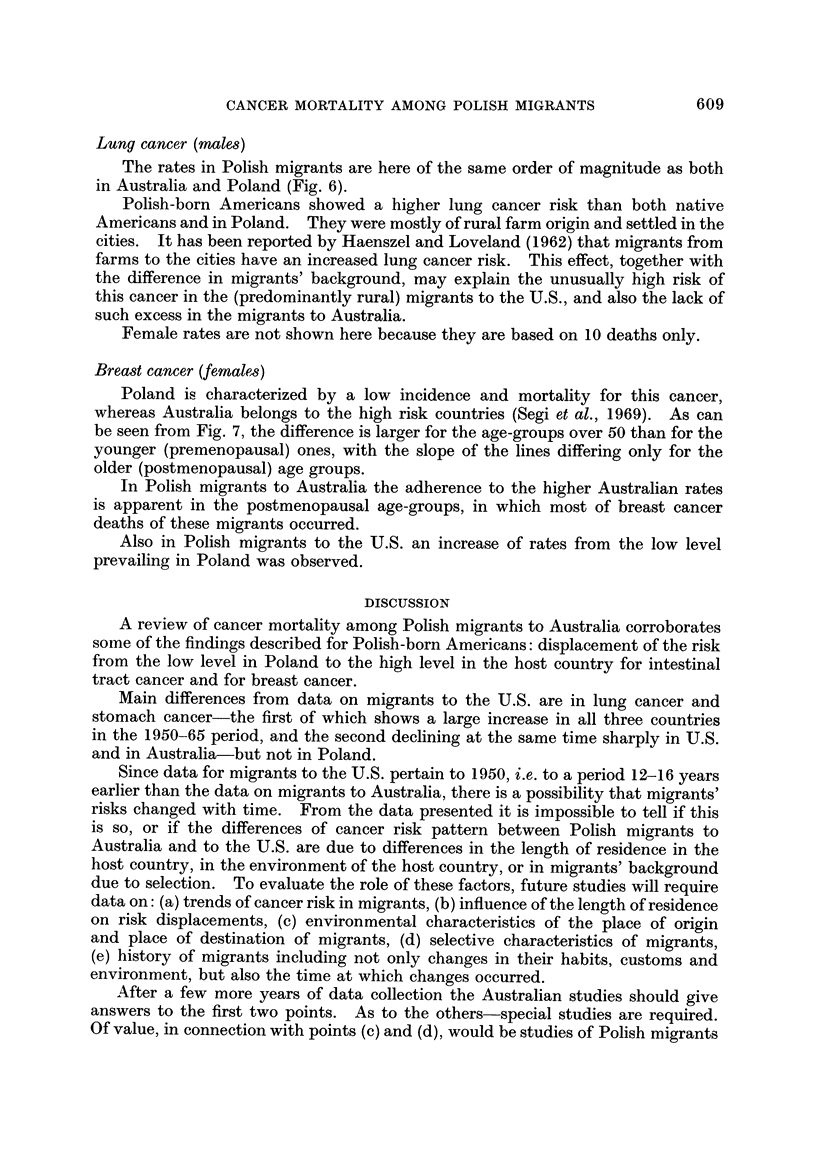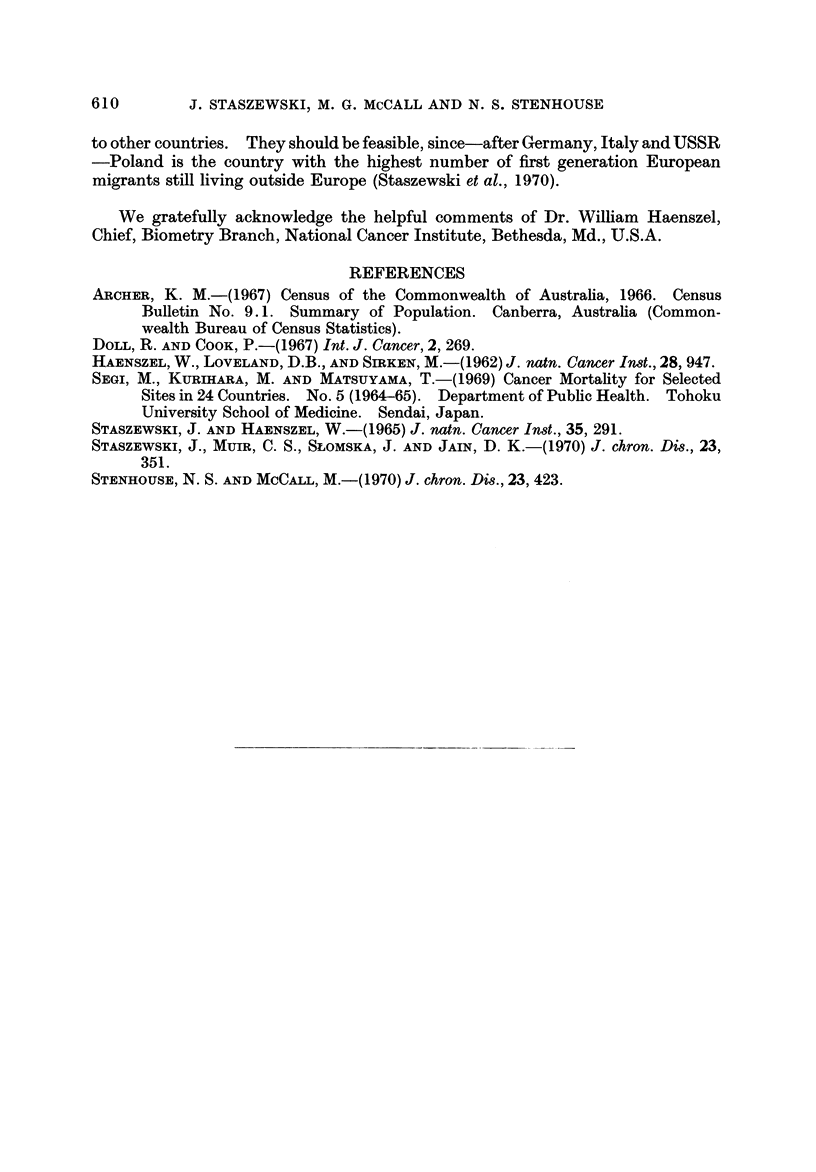# Cancer Mortality in 1962-66 Among Polish Migrants to Australia

**DOI:** 10.1038/bjc.1971.76

**Published:** 1971-12

**Authors:** Jerzy Staszewski, M. G. Mccall, N. S. Stenhouse

## Abstract

The 1962-66 cancer mortality of Polish migrants to Australia is compared with the cancer mortality prevailing in Poland and in Australia. Small numbers compel to limit the analysis to the most frequent cancer sites only.

The main findings are:

(a) Stomach cancer mortality of Polish migrants to Australia is intermediate between the high mortality in Poland and the much lower one in Australia.

(b) Intestinal tract and breast cancer mortality of Polish migrants is displaced upwards, from the low Polish level to the much higher Australian one.

(c) Lung cancer mortality of Polish male migrants does not differ distinctly from the mortality observed both in the country of origin and of adoption of these migrants.

The presented findings are compared with the results of a similar study of Polish migrants to the U.S. Aims for future studies are briefly outlined.


VOL. XXV DECEMBER, 1971 NO. 4

### CANCER MORTALITY IN 1962–66 AMONG POLISH MIGRANTS TO AUSTRALIA

JERZY STASZEWSKI, M. G. McCALL AND N. S. STENHOUSE

*From the Institute of Oncology, Gliwice, Poland, and University of Western Australia, Perth, Australia*

Received for publication June 22, 1971

**SUMMARY.—The 1962–66 cancer mortality of Polish migrants to Australia is compared with the cancer mortality prevailing in Poland and in Australia. Small numbers compel to limit the analysis to the most frequent cancer sites only.**

**The main findings are:**

**(a) Stomach cancer mortality of Polish migrants to Australia is intermediate between the high mortality in Poland and the much lower one in Australia.**
**(b) Intestinal tract and breast cancer mortality of Polish migrants is displaced upwards, from the low Polish level to the much higher Australian one.**
**(c) Lung cancer mortality of Polish male migrants does not differ distinctly from the mortality observed both in the country of origin and of adoption of these migrants.**

**The presented findings are compared with the results of a similar study of Polish migrants to the U.S. Aims for future studies are briefly outlined.**

THE value of epidemiological studies of cancer risk in migrant populations is increasingly appreciated because they may help to relate shifts in disease risks with changes in environmental factors due to migration. Nevertheless, only few data on cancer risk shifts are still published.

With a large part of its population born elsewhere, and with good vital statistics, Australia is the right place for studying migration effects. Such studies were initiated in 1966 and preliminary results for migrants from United Kingdom and from Italy have been published (Stenhouse and McCall, 1970).

At the 1966 census the 62,000 Polish migrants comprised the seventh largest foreign born group in Australia—after United Kingdom (853,000), Italy (267,000), Greece (140,000), Germany (109,000), Netherlands (100,000) and Yugoslavia (71,000) (Archer, 1967).

The preliminary cancer mortality data for Polish migrants to Australia seem interesting enough to present despite the small numbers available. Their value is largely enhanced by the possibility of comparison with mortality of Polish-born Americans (Staszewski and Haenszel, 1965).

Comparing the present data on Polish migrants to Australia with those for Polish migrants to the United States, two qualifications should be kept in mind, firstly the possibility of differing selection of migrants and secondly the effect of time trends, as the American data referred to relates to a period about 10–15 years earlier than that covered by the Australian data.

Whereas the bulk of Polish migration to the United States, which took place before the Second World War (before 1925), consisted of the lowest socio-economic classes, mainly landless peasants, and was due to economic motives, Polish migrants to Australia came mostly after the Second World War, were of higher socio-economic classes than migrants to the U.S., and left Poland mainly because of the war.

This selection of migrants may have some bearing on their cancer risks, because such risks are known to display strong socio-economic and urban-rural gradients. National data from the country of origin may thus be not truly comparable with those for migrants. Analyses in relation to these factors must await the accumulation of additional data. However, where common effects (*e.g.* similar shifts in risk for cancer of some site) are observed in the Polish-born in both Australia and in the U.S., such effects are likely to be significant and not due to selective migration.

TABLE I.—*Deaths from Cancer of Most Frequent Primary Sites in Polish Migrants in Australia*, 1962–66

| Primary site and ICD (1955) number | Sex | Number of deaths |
|---|---|---|
| All sites (140–205) | M | 269 |
| | F | 190 |
| *Where of* | | |
| Stomach (151) | M | 59 |
| | F | 26 |
| Intestinal tract (152–154) | Both | 39 |
| Lung (162, 163) | M | 68 |
| Breast (170) | F | 29 |

The other source of difficulty in interpreting the patterns of cancer mortality in migrants is the possibility of systematic differences or biases in practices for reporting and classifying causes of death. In this respect migrants differ presumably less from the population of their host country than from their country of birth.

The national rates for both Australia and Poland are based on large numbers, which rules out chance variation as an important consideration in their comparison. On the contrary, rates computed for Polish migrants are based on small numbers, as seen from Table I, and are thus subject to sizable chance variation. For that reason little credence should be placed in any single rate for those migrants shown in Fig. 1–7, but attention should be directed to contrasts of the over-all patterns of age-specific rates.

### MATERIAL AND METHODS

The age-specific mortality rates of Polish-born migrants to Australia were available for the 1962–66 period by 10-year age-groups, sex, cause of death and also by the length of residence in Australia (up to 6, 7–18, 19 years and over). However, about three-quarters of cancer deaths fall into one class (those residing in Australia 7–18 years) and thus distinction by the length of residence is not yet of use.

FIG. 1.—Male age-specific mortality rates from cancer, all sites, in Australia and Poland in 1964–65, and in Polish migrants to Australia 1962–66.

Only those sites will be considered separately for which, for the sex-group to be analyzed, more than 25 deaths were observed.

Except for all sites, the data analyzed, as presented in graphs, will be limited to the age-groups 40–79, because, for the sites discussed, only 7 deaths occurred below 40 and 11 at the ages 80 and over.

Polish mortality rates for 1964–65 were computed for 5-year age-groups from the tabulations of deaths by cause, sex and age, obtained yearly from the Central Statistical Office along with corresponding population estimates.

FIG. 2.—Female age-specific mortality rates from cancer, all sites, in Australia and Poland in 1964–65, and in Polish migrants to Australia 1962–66.

Australian data for 1964–65 are taken from the publication by Segi *et al.* (1969). The age-specific rates for Australia, Poland and Polish migrants to Australia are presented in Fig. 1–7.

To compare the over-all mortality rates prevailing in these 3 populations, age-adjustment was necessary. It was done in the following manner. For Australian and Polish mortality the direct method of adjustment was employed, with the " world population " of Segi, as modified by Doll and Cook (1967), as the standard. Only the 40–79 age-groups were used for computation of the age-adjusted rates.

FIG. 3.—Male age-specific mortality rates from stomach cancer in Australia and Poland in 1964–65, and in Polish migrants to Australia 1962–66.

The Australian rates were then taken as 100·0 and Polish rates compared to that base. Data for Polish-born migrants were adjusted by the indirect method, using the 1964–65 age- and sex-specific rates for Australia computed from the data published by Segi *et al.* (1969). The results are presented in Fig. 8.

### RESULTS

*Cancer—all sites*

*In males* up to 75 the mortality by-age curves for all the 3 groups compared were quite parallel, with highest rates observed in Poland, and the rates in Polish

FIG. 4.—Female age-specific mortality rates from stomach cancer, in Australia and Poland in 1964–65, and in Polish migrants to Australia 1962–66.

migrants somewhat lower than in all Australia inhabitants, at least between the ages of 45 and 70 (Fig. 1). If stomach cancer is deleted, the rates in Poland and Australia would be virtually the same, but they would remain lower for Polish migrants.

For ages over 75 the rates continue to increase with age in Australia and in migrants, but stop increasing in Poland.

*In females* up to the age of 75 the rates are higher in Poland than in Australia (Fig. 2). For ages over 75 a similar lack of increase with age is apparent in Polish females as in males. The rates for Polish migrants are higher than in either

FIG. 5.—Age-specific mortality rates from intestinal tract cancer in Australia and Poland in 1964–65, and in Polish migrants to Australia 1962–66. Both sexes combined.

Poland or Australia, except for age groups 40–55. Deletion of stomach cancer would reduce, but not eliminate, these differences.

The lack of the increase with age of cancer mortality rates, observed for both sexes for Poland but not for the other two populations investigated, is probably due to deficiencies of cancer diagnosis and of certification of causes of death in the oldest, and renders the age-groups over 75–80 years not of much value for comparisons. This is an additional reason why the age-groups over 80 have not been used in the analysis by site.

FIG. 6.—Male age-specific mortality rates from lung cancer in Australia and Poland in 1964–65, and in Polish migrants to Australia 1962–66.

Mortality rates of Polish-born Americans were in 1950 of the same order of magnitude as in Polish migrants to Australia in 1962–66.

*Stomach cancer*

As shown in Fig. 3 and 4, for each sex the stomach cancer mortality rates are much higher in Poland than in Australia, even if the mortality-by-age curves are quite parallel.

The rates for Polish migrants fall between these two extremes.

Thus the stomach cancer risk for Polish migrants to Australia differs from that found for Polish-born Americans, whose mortality rates in 1950 were not inter-

FIG. 7.—Female age-specific mortality rates from breast cancer in Australia and Poland in 1964–65, and in Polish migrants to Australia 1962–66.

mediate but similar to those in Poland. This might be due to the fact that the surveys were 10 years apart.

*Intestinal tract cancer*

To decrease chance variation as well as the effects of possible differences in classification of border lesions (recto-sigmoid junction), no subdivision to more detailed sites has been made. This is also justified by the fact that for cancer of both colon and rectum Poland ranks low, when compared with other countries.

Fig. 5 shows that Polish age-specific mortality rates (presented here for both sexes combined to secure larger numbers) are consistently lower than the Australian ones, and the curves by age are quite parallel. The rates for Polish migrants

FIG. 8.—Age-adjusted cancer mortality rates by site and sex in Australia and Poland in 1964–65, and in Polish migrants to Australia 1962–66. (For methods of adjustment see text.)

follow rather the pattern of their country of adoption, as was found in migrants to the U.S.

The female age-adjusted intestinal tract cancer mortality rates in Australia and in Polish migrants to Australia are similar, and much lower in Poland (Fig. 8). For males the age-adjusted rates are lower in Polish migrants to Australia than the rates prevailing in their country of adoption, even if higher than in Poland. This sex differential in migrant risk shifts may be due to chance variation, as the rates are based on small numbers (Table I).

*Lung cancer (males)*

The rates in Polish migrants are here of the same order of magnitude as both in Australia and Poland (Fig. 6).

Polish-born Americans showed a higher lung cancer risk than both native Americans and in Poland. They were mostly of rural farm origin and settled in the cities. It has been reported by Haenszel and Loveland (1962) that migrants from farms to the cities have an increased lung cancer risk. This effect, together with the difference in migrants' background, may explain the unusually high risk of this cancer in the (predominantly rural) migrants to the U.S., and also the lack of such excess in the migrants to Australia.

Female rates are not shown here because they are based on 10 deaths only.

*Breast cancer (females)*

Poland is characterized by a low incidence and mortality for this cancer, whereas Australia belongs to the high risk countries (Segi *et al.*, 1969). As can be seen from Fig. 7, the difference is larger for the age-groups over 50 than for the younger (premenopausal) ones, with the slope of the lines differing only for the older (postmenopausal) age groups.

In Polish migrants to Australia the adherence to the higher Australian rates is apparent in the postmenopausal age-groups, in which most of breast cancer deaths of these migrants occurred.

Also in Polish migrants to the U.S. an increase of rates from the low level prevailing in Poland was observed.

### DISCUSSION

A review of cancer mortality among Polish migrants to Australia corroborates some of the findings described for Polish-born Americans: displacement of the risk from the low level in Poland to the high level in the host country for intestinal tract cancer and for breast cancer.

Main differences from data on migrants to the U.S. are in lung cancer and stomach cancer—the first of which shows a large increase in all three countries in the 1950–65 period, and the second declining at the same time sharply in U.S. and in Australia—but not in Poland.

Since data for migrants to the U.S. pertain to 1950, *i.e.* to a period 12–16 years earlier than the data on migrants to Australia, there is a possibility that migrants' risks changed with time. From the data presented it is impossible to tell if this is so, or if the differences of cancer risk pattern between Polish migrants to Australia and to the U.S. are due to differences in the length of residence in the host country, in the environment of the host country, or in migrants' background due to selection. To evaluate the role of these factors, future studies will require data on: (a) trends of cancer risk in migrants, (b) influence of the length of residence on risk displacements, (c) environmental characteristics of the place of origin and place of destination of migrants, (d) selective characteristics of migrants, (e) history of migrants including not only changes in their habits, customs and environment, but also the time at which changes occurred.

After a few more years of data collection the Australian studies should give answers to the first two points. As to the others—special studies are required. Of value, in connection with points (c) and (d), would be studies of Polish migrants to other countries. They should be feasible, since—after Germany, Italy and USSR —Poland is the country with the highest number of first generation European migrants still living outside Europe (Staszewski *et al.*, 1970).

We gratefully acknowledge the helpful comments of Dr. William Haenszel, Chief, Biometry Branch, National Cancer Institute, Bethesda, Md., U.S.A.

### REFERENCES

ARCHER, K. M.—(1967) Census of the Commonwealth of Australia, 1966. Census Bulletin No. 9.1. Summary of Population. Canberra, Australia (Commonwealth Bureau of Census Statistics).
DOLL, R. AND COOK, P.—(1967) *Int. J. Cancer*, **2**, 269.
HAENSZEL, W., LOVELAND, D.B., AND SIRKEN, M.—(1962) *J. natn. Cancer Inst.*, **28**, 947.
SEGI, M., KURIHARA, M. AND MATSUYAMA, T.—(1969) Cancer Mortality for Selected Sites in 24 Countries. No. 5 (1964–65). Department of Public Health. Tohoku University School of Medicine. Sendai, Japan.
STASZEWSKI, J. AND HAENSZEL, W.—(1965) *J. natn. Cancer Inst.*, **35**, 291.
STASZEWSKI, J., MUIR, C. S., SŁOMSKA, J. AND JAIN, D. K.—(1970) *J. chron. Dis.*, **23**, 351.
STENHOUSE, N. S. AND McCALL, M.—(1970) *J. chron. Dis.*, **23**, 423.